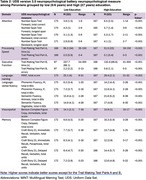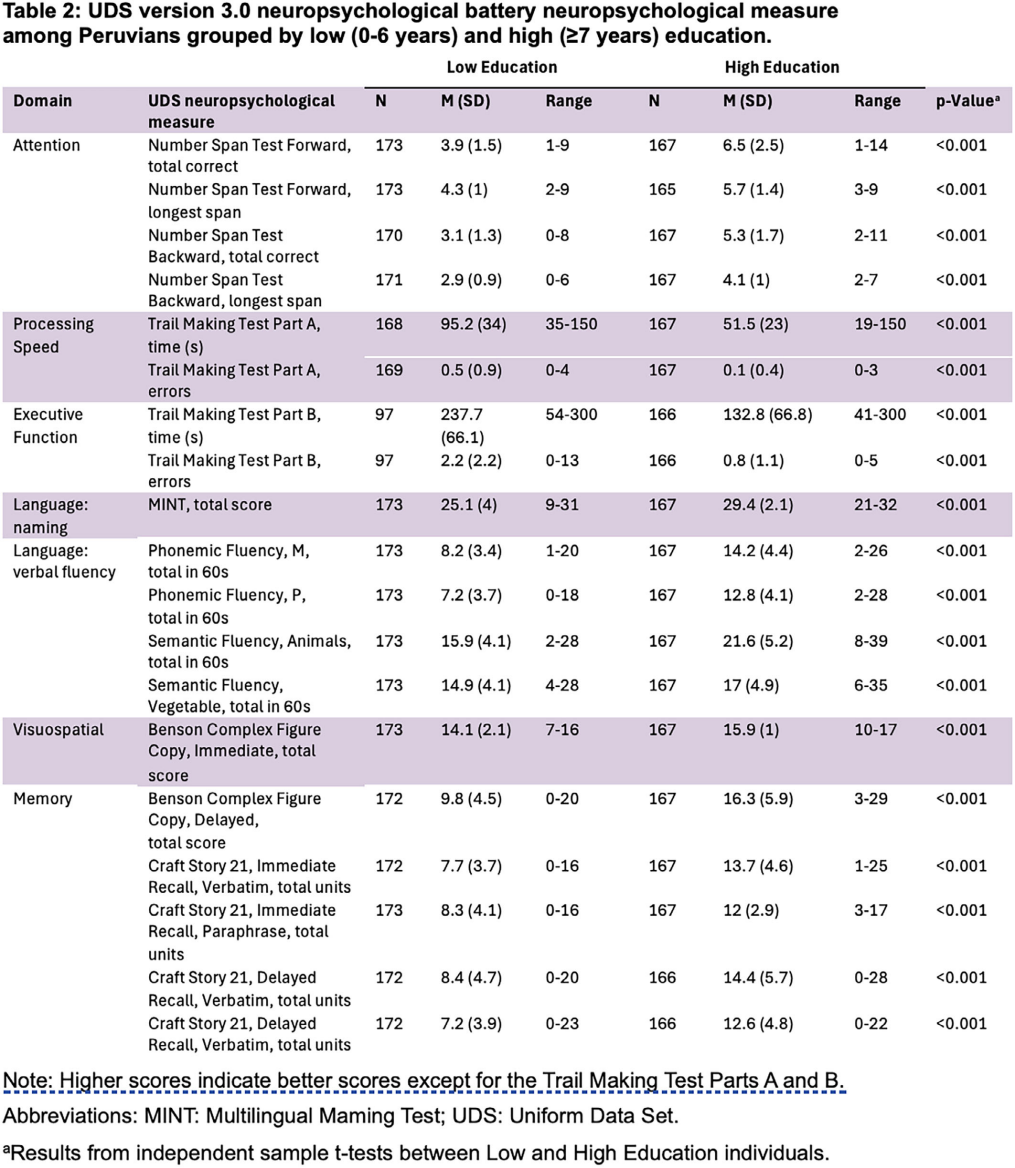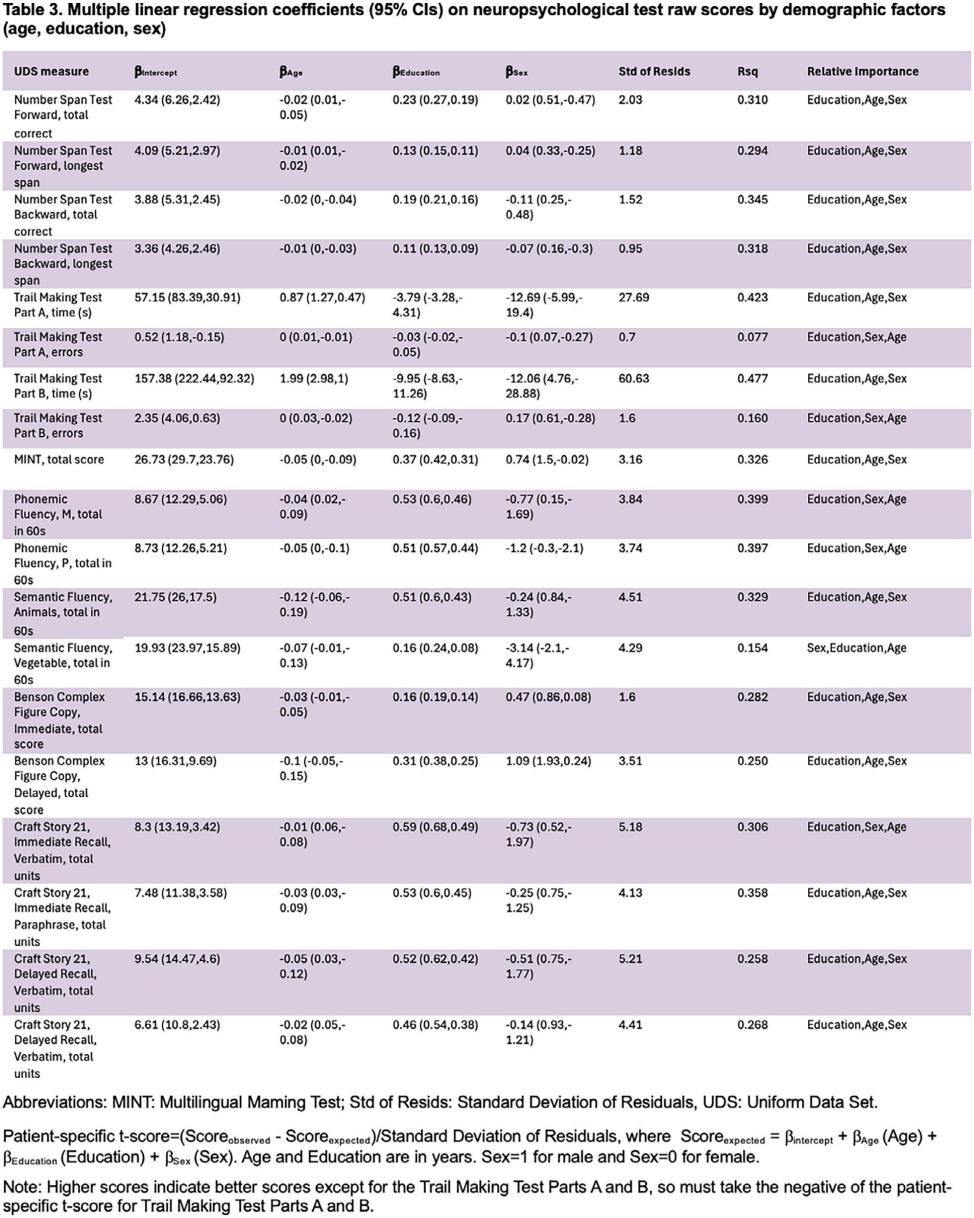# Demographically‐Adjusted Normative Data Among Peruvians with Diverse Education Levels for Version 3 of the Alzheimer's Disease Centers Neuropsychological Test Battery in the Uniform Data Set

**DOI:** 10.1002/alz70857_104880

**Published:** 2025-12-25

**Authors:** Gregory Brown, Diego Bustamante‐Paytan, Maria Fe Albujar‐Pereira, José Carlos Huilca, Katherine Agüero, Graciet Verástegui, Zadith Yauri, Pamela Bartolo, Daniela Bendezu, Rosa Montesinos, Agustin Ibanez, Nilton Custodio

**Affiliations:** ^1^ Instituto Peruano de Neurociencias, Lima, Lima, Peru; ^2^ University of California, San Francisco, San Francisco, CA, USA; ^3^ Equilibria, Lima, Lima, Peru; ^4^ Universidad de San Martín de Porres, Facultad de Medicina, Centro de Investigación del Envejecimiento, Lima, Lima, Peru; ^5^ Unidad de Investigación y Docencia, Equilibria, Lima, Peru., Lima, Lima, Peru; ^6^ Latin American Brain Health Institute (BrainLat), Universidad Adolfo Ibañez, Santiago, Chile; ^7^ Global Brain Health Institute (GBHI), University of California San Francisco (UCSF); & Trinity College Dublin, Dublin, Ireland

## Abstract

**Background:**

The diagnosis of Alzheimer's disease requires standardized neuropsychological assessments, adapted and validated for each community. Demographically adjusted normative data are critical for accurate evaluations. The Uniform Data Set (UDS) neuropsychological battery, widely used in the National Alzheimer's Coordinating Center (NACC), is a multi‐domain assessment for early dementia detection and tracking. Its standardized design supports cross‐site comparability and longitudinal monitoring. However, the UDS has not been validated for populations with low education levels, such as those in Peru.

**Methods:**

We recruited 340 healthy participants (ages 43‐79, 70% female), strategically balanced by education level: low (0‐6 years, *n* = 173) and high (≥7 years, *n* = 167). Participants underwent UDS battery, including number span, trail making, image naming, verbal fluency, figure copying, and story recall. Group comparisons were made using t‐tests. Normative values were generated through linear regression, incorporating age, education, and sex as predictors.

**Results:**

Participants were well‐matched for age (*p* = 0.970) and sex (*p* = 0.904) between the low and high education groups. Education emerged as the primary influential factor across all measures, except semantic fluency: vegetables. Age emerged as the second most significant predictor for 68% of the assessments. The largest effect sizes of low education (|Cohen's d|>1) were on backwards digit span, time for trails making A & B, image naming, phonemic fluency, and immediate figure copy and story recall.

**Conclusion:**

This is the first comprehensive, demographically stratified normative cognitive data for Peruvian adults. The generated tables are valid for all education levels in Peruvian individuals ranging from 43‐79 years of age. Individual t‐scores can be determined by calculating the expected score [Score_expected_ = β_Intercept_ + β_Age_ (Age) + β_Education_ (Education) + β_Sex_ (Sex)] and then subtract Score_expected_ from the measured score and divide by the standard deviation of the residuals. We found education to most effect the domains of processing speed, working memory, memory encoding, and word finding. Culturally appropriate normative data is essential for accurate early detection of cognitive impairments. Future work is needed to determine if this data is accurate for other Spanish‐speaking South America countries and for patients with cognitive decline and dementia.